# Chronic hypokalemia: The hidden connection to early systemic lupus

**DOI:** 10.1097/MD.0000000000046549

**Published:** 2026-02-06

**Authors:** Chetan Khoshoo, Kavya Seenahalli Thimmaiah, Anoop Marilingegowda, Bangalore Raja Shivakumar, Parashuram Bhavimani, Avinash Hanbe Rajanna

**Affiliations:** a Department of General Medicine, Dr B.R. Ambedkar Medical College, Bengaluru, Karnataka, India; b Department of General Medicine, Bangalore Medical College and Research Institute, Bengaluru, Karnataka, India; c Department of General Medicine, Shri Atal Bihari Vajpayee Medical College and Research Institution, Bengaluru, Karnataka, India.

**Keywords:** autoimmune disorder, chronic hypokalemia, corticosteroids, electrolytes balance disorder, systemic lupus erythematosus

## Abstract

**Rationale:**

Currently, hypokalemia is managed with potassium supplements; however, there are some rare cases where it can hint towards autoimmune diseases such as systemic lupus erythematosus (SLE). The purpose of this case is to bring attention to the diagnostic difficulty of SLE in the absence of typical mucocutaneous features.

**Patient concerns:**

The symptoms included weakness and swelling of the legs. The patient’s history revealed persistent chronic hypokalemia over a 4-year period, during which she was treated with potassium supplements, yet without any primary cause being identified.

**Diagnoses:**

A comprehensive evaluation found both hematological and renal abnormalities. Notably, despite the lack of classic dermatological signs of lupus, she scored 23 on the European League Against Rheumatism/American College of Rheumatology scoring system. She also screened positive for autoimmune disorders with a significant elevated antinuclear antibody profile including high lupus-specific autoantibodies. Subsequent bone marrow evaluation ruled out other hematological malignancies. Considering all the findings, she was diagnosed with SLE.

**Interventions:**

She was treated with corticosteroid therapy, consistent with her diagnosis and forming part of the prescribed standard immunosuppressive treatment regimen.

**Outcomes:**

The patient demonstrated significant clinical improvement with regard to her symptoms and her potassium levels. She remained stable in further follow-up.

**Lessons:**

This case illustrates the importance of very active investigation of the cause of persistent hypokalemia and a multidisciplinary collaboration. Autoimmunity should be considered in chronic electrolyte derangements, particularly in the presence of some blood or kidney problem. SLE is one of the conditions that can be diagnosed and treated early for better clinical outcome.

## 1. Introduction

Hypokalemia (HK) occurs when serum potassium levels are lower than normal (<3.5 mEq/L). It is a rare and frequently clinically undiagnosed condition, and its diagnosis is established using serum electrolytes. Patients with hypokalemia may have systemic conditions due to nonspecific symptoms. Hypokalemia has several causes, but replacement therapy is the most common treatment. In this situation, professionals neglect deeper systemic ailments that would provide the final diagnosis and cure owing to their simplicity of treatment. Clinicians must identify systemic diseases to quickly make a final diagnosis. A middle-aged woman with chronic hypokalemia for 4 years was diagnosed with early systemic lupus erythematosus (SLE) after extensive testing.

## 2. Case report

A middle-aged female patient, a housewife, was evaluated at our hospital with a 3-month history of generalized weakness, followed by swelling of both lower limbs.

The patient was asymptomatic until 3 months ago, when she developed generalized weakness, lower limb edema, and decreased appetite. She was admitted to another hospital for antibiotic and supportive care for typhoid fever-related pancytopenia. Her lower limbs were swollen for a month when she was hospitalized. No dyspnea or stomach distension was observed.

She had no history of fever, weight loss, or rash.

The patient is a known case of hypothyroid for 5 years, on Tab Thyronorm (levothyroxine) 25 µg 1 OD with reasonable control. The last thyroid evaluation performed 3 months before admission was unremarkable.

She had been admitted 4 years back to another hospital for progressive weakness of the lower limbs followed by the upper limbs 4 years ago, where she was finally diagnosed with hypokalemic periodic paralysis. She was advised to consume syrup potassium chloride 15 mL along with 1 glass of water for 1 year, following which she used to take the syrup only when she was feeling weak. The patient recovered uneventfully.

The patient had no other relevant medical history. The patient had no known family history of autoimmune disorders or other chronic systemic illnesses. Lifestyle factors including diet and occupation were unremarkable.

On general examination, she was averagely built with a BMI of 19.6 kg/m^2^ and had pallor and bilateral pitting pedal edema up to the knee. Her pulse was 106/min and, regular. Her blood pressure was 90/60 mm/Hg, and her JVP increased by approximately 8 cm in H_2_O. All other findings were unremarkable. Systemic examination of abdomen showed hepatosplenomegaly.

**Figure 1.**
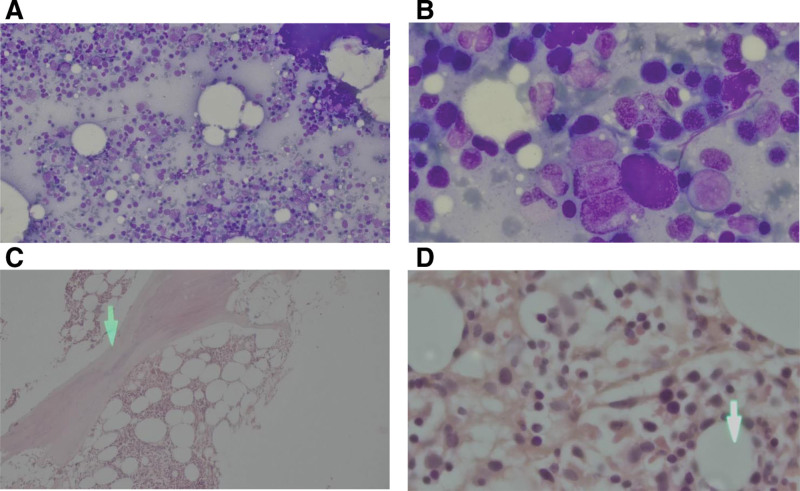
Bone marrow aspiration and biopsy of the patient as described in Table 1. (A) Bone marrow aspiration under low magnification (40×). (B) Bone marrow aspiration under high magnification (100×). (C) Bone marrow biopsy under low magnification (40×) arrow depicting Hyalinization of the bone marrow. (D) Bone marrow biopsy under high magnification (100×) arrow highlighting increased fat vacuoles in bone marrow suggestive of hypocellular marrow.

On admission, her initial investigations showed pancytopenia in complete blood counts. Bone marrow biopsy and aspiration were performed for further evaluation of pancytopenia, suggesting hypocellular marrow with mild erythroid hyperplasia showing a micro-normoblastic maturation pattern as depicted in Figure 1.

**Table 1:** Baseline laboratory investigations on admission including complete blood count and metabolic panel.

Clinical tests/parameter	Initial values	Reference range
CBC		
Hb	**6.4**	11.5–16 g/dL
TLC	**3930**	4000–11,000 cells/cumm
Neutrophils	68.1	40–70%
Lymphocytes	25	20–45%
Eosinophils	0.7	1–6%
Monocytes	3.6	2–10%
Basophils	0.2	0–1%
Platelet count	**0.72**	1.5–4.5 lakhs/cumm
RBC	**2.53**	3.9–5 mili/cumm
PCV	**20.1**	37–47%
MCV	79.4	78–100 fL
MCH	25.2	25–32 pg
MCHC	31.7	32–35%
ESR	**25**	0–20 mm/h
Serum electrolytes		
Sodium	141.3	135–145 mmol/L
Potassium	**3.2**	3.5–5 mmol/L
Chloride	**125.2**	97–108 mmol/L
Renal function test		
Serum urea	**4.6**	15–45 mg/dL
Serum creatinine	0.6	0.6–1.2 mg/dL
C-reactive protein	**24.3**	Up to 5 mg/dL
Ultrasound abdomen and pelvis	Both kidneys normal in size and echotexture, hepatomegaly and splenomegaly	
Bone marrow aspiration and biopsy	Features are suggestive of hypocellular marrow with mild erythroid hyperplasia showing a micronormoblastic pattern of maturation	
Urine routine and microscopy		
pH	7.0	5–9 pH
Albumin	**Nil**	
Sugar	Nil	
Pus cells	1–2	
Epithelial cells	1–2	
RBC	**Nil**	
Serum magnesium	**1.4**	1.6–2.3 mg/dL

Bold values indicate results outside the normal reference range (abnormal values).

CBC = complete blood count; ESR = erythrocyte sedimentation rate; Hb = hemoglobin; MCH = mean corpuscular hemoglobin; MCHC = mean corpuscular hemoglobin concentration; MCV = mean corpuscular volume; PCV = packed cell volume; RBC = red blood cell (count); TLC = total leukocyte count.

**Figure 2.**
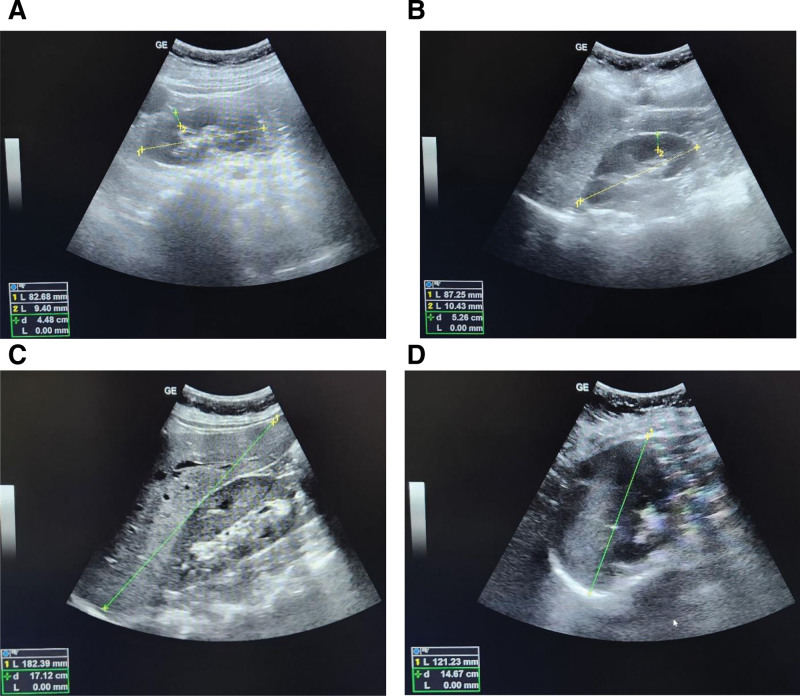
Ultrasonography images of the patient as described in Table 1. (A) Ultrasonography images of left kidney (8.27 cm showing normal echotexture). (B) Ultrasonography images of right kidney (8.72 cm showing normal echotexture). (C) Ultrasonography images of liver showing hepatomegaly (18.2 cm). (D) Ultrasonography images of spleen showing splenomegaly (12.1 cm).

The patient was further evaluated and all initial investigations, along with ultrasonography images of the patient are as under given in Table 1 and Figure 2 respectively.

A nephrologist’s opinion was sought, and she was worked up further for hypokalemia, which showed serum calcium of 6.4 mg/dL, vitamin D_3_ – 13 ng/mL, antinuclear antibody (ANA) screening to be 3+ with a positive homogenous nucleoplasm pattern, 24 hours urine creatinine to be 417 mg/24 h, and 24 hours urine protein to be 726 mg/d. All the other investigations ordered by the nephrologist is as given in Table 2.

**Table 2:** Renal function and electrolyte parameters as evaluated by the nephrology team.

Clinical tests/parameter	Initial values	Reference range
ABG		
pH	7.361	7.35–7.45
pCO_2_	21.0	35–48 mmol/L
pO_2_	82.3	83–108 mmol/L
HCO_3_^−^	**17.6**	18–23 mmol/L
Lactate	1.76	0.5–2.2 mmol/L
Urine osmolality	**223**	300–800 mOsmol/kg
Serum osmolality	292	
Urine electrolytes		
Urine sodium	**93**	
Urine potassium	**12.4**	
Urine chloride	**106**	
Urine calcium	3.9 mg/dL	
Urine creatinine	22.2 mg/dL	
Urine calcium/creatinine ratio	**0.176**	Expected < 0.2
Urine citrate (24 h)	**170.67**	250–1160 mg/24 h
Urine phosphorous (24 h)	**183.3**	400–1300 mg/24 h
TTKG	**5.237**	
Serum calcium	**6.4**	8.6–10.3 mg/dL
Serum phosphorous	**1.2**	2.5–4.5 mg/dL
Vitamin D_3_	**13**	30–100 ng/mL

Bold values indicate results outside the normal reference range (abnormal values).

ABG = arterial blood gas, TTKG = trans tubular potassium gradient.

Rheumatologist’s opinion was sought and the investigations advised by him as in Table 3.

**Table 3:** Autoimmune screening and immunological markers as advised by rheumatologist.

Clinical tests/parameter	Initial values	Reference range
Complement		
C3 serum	**34.22**	90–180 mg/dL
C4 serum	**<4.00**	10–40 mg/dL
24 h urine protein	**726 mg/d**	0–150 mg/d
Procalcitonin	0.07 ng/mL	0–0.15 ng/mL
Total CPK	25	24–190 U/L
Coombs test		
Direct	**Negative**	Negative
Indirect	**Negative**	Negative
Blood culture (repeated)	Culture yields no growth after incubation for 1 wk at 37°C	

Bold values indicate results outside the normal reference range (abnormal values).

**Figure 3.**
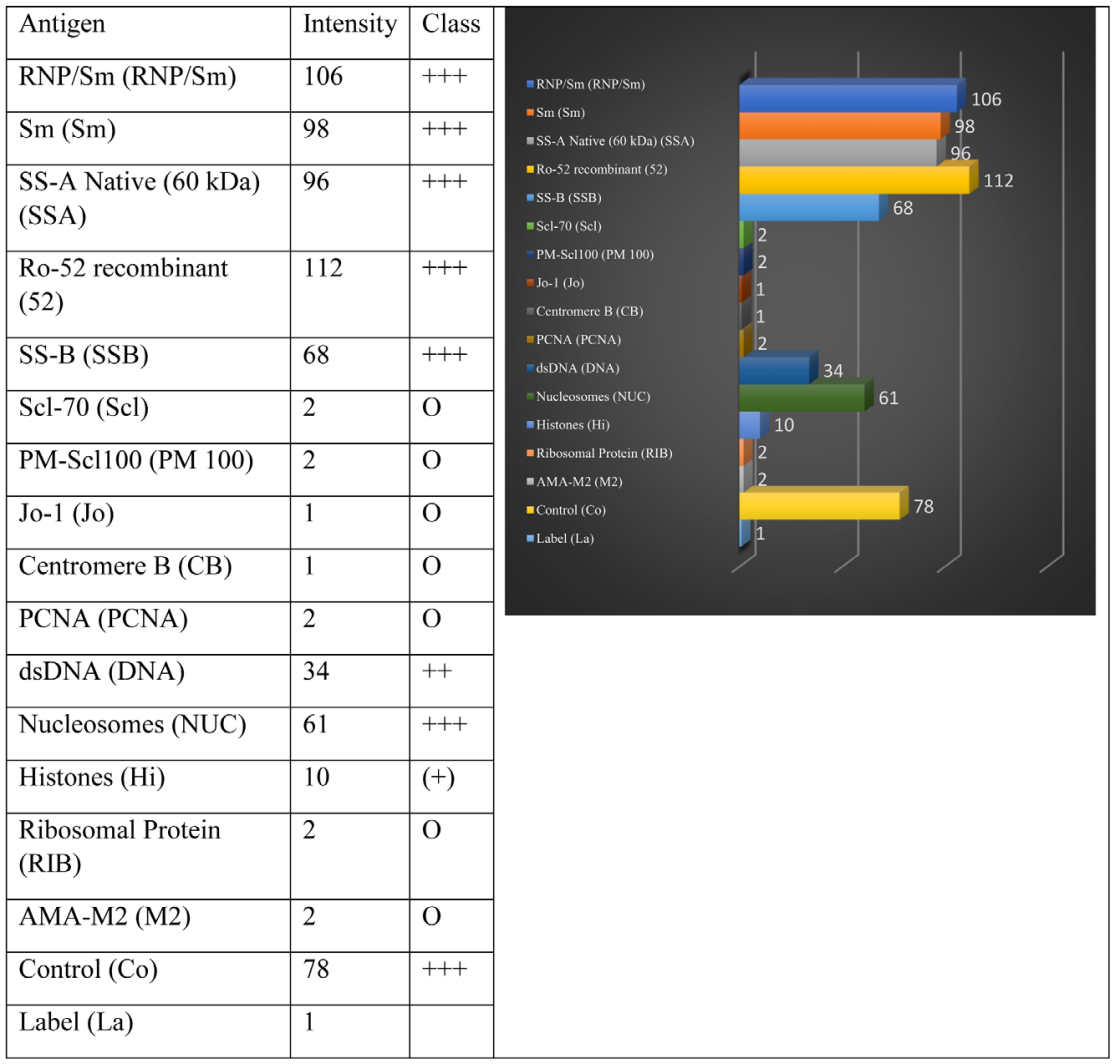
ANA immunoblot assay showing positivity for RNP/Sm, Sm, SSA, Ro-52, SSB, and nucleosome antibodies. ANA = antinuclear antibody.

The ANA profile (as given in Fig. 3) was 3+ positive for RHP/Sm, Sm, native SSA, Ro-52, SSB, and nucleosomes.

Rheumatologist’s opinion was obtained and ANA profile was suggestive of SLE. She was initiated on Tab Wysolone (Prednisolone) 40 mg once daily for 8 weeks and which was tapered slowly by 10 mg every week and was given 5 mg alternate days for 5 days. Patient was also given vitamin D, calcium, phosphorous and magnesium supplementation. Patient is progressing well and is currently healthy.

## 3. Follow-up and outcomes

**Figure 4.**
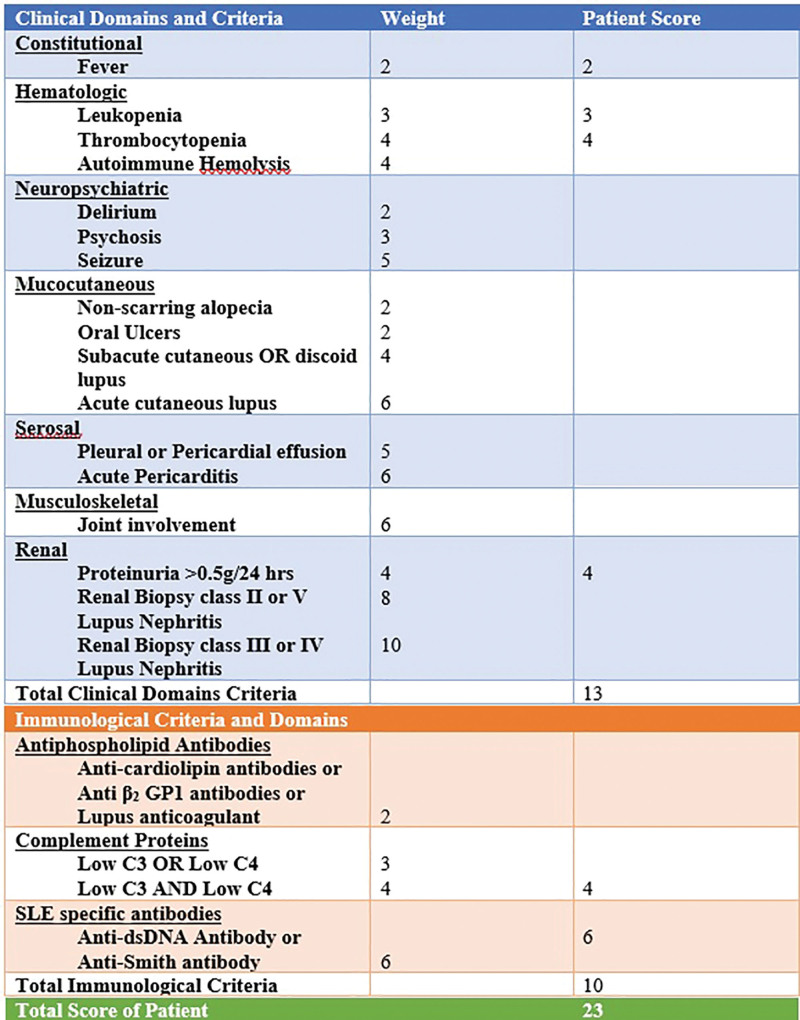
Patients score as per EULAR/ACR Criteria.^[1]^ ACR = American College of Rheumatology, EULAR = European League Against Rheumatism.

According to 2019 European League Against Rheumatism/American College of Rheumatology Criteria as elucidated in Figure 4, she had a total of 13 points from clinical criteria: −2 from constitutional, 7 from hematologic and 4 from renal criteria. Her immunological criteria included a total weight of 10: −4 from complements and 6 from SLE-specific antibodies. With a total weight of 23, she was a diagnosed with SLE as described in Figure 4.

A nephrologist’s opinion was taken for renal biopsy which would be conclusive in view of LN. However, as the patient was in the initial nephritic range of proteinuria and unwilling to undergo a renal biopsy, it was deferred at the moment. The patient was followed up and the follow-up investigations after 2 months are as under in Table 4. The patient was symptomatically better with no new complaints.

**Table 4:** Follow up laboratory investigations after 1 mo and 2 mo respectively.

Clinical tests/parameters	Follow up 1st mo	Follow up 2nd mo	Reference range
CBC			
Hb	**8.0**	11.9	11.5–16 g/dL
TLC	6240	**3250**	4000–11,000 cells/cumm
Neutrophils	77.1	56.3	40–70%
Lymphocytes	17.6	34.8	20–45%
Eosinophils	0.5	1.5	1–6%
Monocytes	4.7	7.4	2–10%
Basophils	0.1	0	0–1%
Platelet count	1.35	1.68	1.5–4.5 lakhs/cumm
RBC	**2.89**	4.21	3.9–5 mili/cumm
PCV	**24**	38.4	37–47%
MCV	83.3	91.2	78–100 fL
MCH	27.6	28.3	25–32 pg
MCHC	33.2	31	32–35%
ESR	18	16	0–20 mm/h
Serum electrolytes			
Sodium	**129.2**	136	135–145 mmol/L
Potassium	3.5	4.11	3.5–5 mmol/L
Chloride	**109**	**112**	97–108 mmol/L
Renal function test			
Serum urea	18.8	**11.88**	15–45 mg/dL
Serum creatinine	0.5	0.4	0.6–1.2 mg/dL
Urine routine and microscopy			
pH	7.0	7.0	5–9 pH
Albumin	**Nil**	Negative	
Sugar	Nil	Nil	
Pus cells	1–2	4–6	
Epithelial cells	1–2	1–2	
RBC	Nil	4	
Serum magnesium	**1.6**	1.8	1.6–2.3 mg/dL
Serum calcium	**7.6**	9.1	8.6–10.3 mg/dL
Serum phosphorous	**1.8**	2.6	2.5–4.5 mg/dL
Complement			
C3 serum	**68.3**	**88.2**	90–180 mg/dL
C4 serum	**6.2**	**9.2**	10–40 mg/dL

Bold values indicate results outside the normal reference range (abnormal values).

CBC = complete blood count; ESR = erythrocyte sedimentation rate; Hb = hemoglobin; MCH = mean corpuscular hemoglobin; MCHC = mean corpuscular hemoglobin concentration; MCV = mean corpuscular volume; PCV = packed cell volume; RBC = red blood cell (count); TLC = total leukocyte count.

## 4. Timeline

The timeline of the above case is as listed in Table 5.

**Table 5:** Chronological clinical events in patient’s case timeline.

Date	Event
4-yr ago	First episode of hypokalemic periodic paralysis diagnosed
3-mo ago	Developed generalized weakness, lower limb edema, and appetite loss
Initial admission	Investigations showed pancytopenia and chronic hypokalemia
Post-investigations	Diagnosed with early SLE based on EULAR/ACR 2019 criteria
4-wk after treatment	Symptomatic improvement on corticosteroids

ACR = American College of Rheumatology, EULAR = European League Against Rheumatism, SLE = systemic lupus erythematosus.

## 5. Discussion

Chronic hypokalemia, defined as a sustained reduction in serum potassium levels, can lead to significant disturbances in neuromuscular, cardiovascular, and renal physiology, often resulting in profound clinical consequences if unrecognized or untreated. Emerging evidence highlights an underappreciated association between persistent hypokalemia and SLE, particularly due to autoimmune-mediated renal tubular dysfunction such as distal or type 4 renal tubular acidosis, which results in excessive urinary potassium wasting.^[2,3]^ Population-based studies further demonstrate that electrolyte abnormalities, including potassium disturbances, are relatively common in patients with SLE and may reflect early renal involvement before overt nephritis develops.^[4]^ In addition to autoimmune tubular injury, factors such as inadequate dietary intake, hormonal dysregulation, and medication-induced renal potassium losses contribute to the pathogenesis of electrolyte imbalance and play an important role in determining potassium homeostasis in systemic illnesses.^[5]^ Collectively, these findings support the concept that chronic hypokalemia may serve as an early clinical clue to underlying autoimmune disease, including SLE, particularly when accompanied by renal or hematologic abnormalities.

Potassium is a major intracellular ion and one of the most essential nutrients required for normal physiology. Potassium homeostasis is maintained by balancing potassium intake in the diet with excretion, and by altering its distribution around the body.^[6]^

Most of the cellular K^+^ in the body (~50 mmol/kg, 98%) resides primarily in skeletal muscles (3000 mmol), red blood cells (300 mmol), and the liver (200 mmol).^[7]^

Hypokalemia is classified as mild (3–3.5 mmol/L), moderate (2.5–3 mmol/L) and severe (if <2.5 mmol/L).^[8]^

In clinical practice and hospitalization, hypokalemia is common. Approximately 14% to 40% of hospitalized patients have hypokalemia, with 5% having potassium levels below 3.0 mmol/L. Hypokalemia risk increases with female sex, younger age, high estimated glomerular filtration rate, and baseline diuretic use. The Na^+^–K^+^–ATPase pump controls K^+^. It pumps Na^+^ out of the cell and pulls K^+^ in, providing a higher intracellular K^+^ gradient than extracellular K^+^ to maintain potential differences between membranes in some cells.^[8]^

Few human physiological functions have a more constant and persistent circadian rhythm than urinary potassium excretion. The circadian rhythm of renal potassium excretion adjusts slowly over several days to the local day–night cycle after transatlantic plane travel. Meals cause substantial, transitory increases in renal potassium excretion due to rapid changes in the distal nephron active potassium secretion and reabsorption. These reactionary reactions are placed on a predictive increase in these transport systems during mealtimes. This predictor of potassium homeostasis uses tubule cell circadian clocks synchronized with the central clock of the brain.^[9]^

In proximal convoluted tubule, potassium is reabsorbed after glomerular filtration. Water and sodium are passively transported to the distal convoluted tubule. Principal- and alpha-intercalated cells affect late distal convoluted tubule and collecting duct potassium. Through the renal outer medullary potassium channel, the principal cells release potassium into the lumen and reabsorb sodium through the epithelial sodium channel. Only 25% of the individuals with hypokalemia had potassium symptoms. The symptom intensity depends on the duration of potassium deficiency and hypokalemia. Weakness, fatigue, muscle cramps, and myalgia are the early signs of hypokalemia. Hypokalemia alters circulation and electrocardiogram, increasing the risk of arrhythmia.^[10]^

Hypokalemia can be caused by 2 conditions: renal potassium wasting diseases such as diabetic ketoacidosis, Cushing’s syndrome, primary hyperaldosteronism, Bartter syndrome, Gitelman syndrome, hypomagnesemia, and renal tubule acidosis, as well as loop and thiazide diuretics, which cause kidney K loss, and extra-renal excretion or transient shifting conditions. Measurements of urine potassium excretion, blood pressure, and acid-base levels can help diagnose hypokalemia.^[11]^

If the etiology of hypokalemia is unclear, a 24 hours urine collection for K^+^ is helpful. If hypokalemia is due to GI loss, the kidneys will preserve K^+^ and the 24 hours urine K^+^ level is <30 mEq. In patients with renal loss of K^+^, 24 hours urine K^+^ is ≥30 mEq.^[4,5,12,13]^

Interestingly, recent evidence has identified a clinically relevant association between chronic hypokalemia and SLE, primarily through autoimmune-mediated renal tubular injury such as distal or type 4 renal tubular acidosis, which results in inappropriate urinary potassium wasting.^[2,3]^ Because SLE frequently affects the kidneys, both glomerular and tubular involvement can impair potassium homeostasis, placing patients at increased risk of sustained hypokalemia even before overt nephritis becomes clinically apparent.^[4]^ Furthermore, chronic kidney disease – commonly encountered in the course of lupus nephritis – can exacerbate disturbances in potassium excretion, compounding the risk of chronic hypokalemia in affected individuals.^[13]^ These findings underscore the importance of recognizing electrolyte imbalances as potential early indicators of renal involvement in SLE.^[3]^

Current research indicates that chronic hypokalemia may contribute to the development and progression of SLE. Potassium is essential for maintaining normal immune function, and chronic hypokalemia has been shown to impairs T-cell signaling and promotes autoimmunity.^[12]^ Potassium deficiency can also lead to oxidative stress and inflammation, which play a role in the pathogenesis of SLE.^[14]^

SLE is a multiorgan disease with renal involvement. Interstitial involvement, such as renal tubular acidosis (RTA), is rarer than glomerular involvement owing to potassium imbalance in almost all cases.^[3]^

SLE is a multisystem autoimmune disorder in which renal involvement is common and often clinically significant. Although glomerular disease is the predominant pattern in lupus nephritis, interstitial and tubular involvement – particularly renal tubular acidosis (RTA) – is increasingly recognized and may occur either before or after the diagnosis of SLE.^[2,13]^ Distal renal tubular acidosis (type 1) represents the most frequently reported tubular abnormality in SLE and is characterized by impaired urinary acidification resulting from autoimmune-mediated tubular dysfunction. This may occur with defects such as decreased hydrogen ion secretion, impaired urinary concentration, or disturbances in renin–aldosterone signaling, contributing to features such as hyporeninemic hypoaldosteronism and clinically significant hypokalemia.^[3,13]^ Recognition of these tubular manifestations is essential, as they can precede overt glomerular disease and provide early clues to underlying autoimmune renal injury.

As in the general population, sustained HK in SLE and lupus nephritis (LN) may be due to extrarenal potassium loss, such as diarrhea or renal loss. Renal potassium wasting may occur in patients with SLE and LN due to exposure to diuretics and corticosteroids, which are commonly used in the management of LN. A syndrome of idiopathic HK was revealed in 20 of 403 (5%) patients within the American LN population, and proved to be distinct from the RTA that occurs in LN. Furthermore, it is associated with a distinct autoantibody pattern. It has been speculated that idiopathic HK is the result of a novel target of autoimmunity in LN, affecting renal tubular potassium transport.^[15]^

## 6. Implications and future research

Given the complex and multifaceted nature of the connection between chronic hypokalemia and SLE, additional studies are warranted to further explore this intriguing and clinically relevant association.

Further research is needed to elucidate the precise pathways and mechanisms that link electrolyte imbalances, such as chronic hypokalemia, to the onset of autoimmune disorders and other diseases.

## 7. Key takeaways

Profound hypokalemia is a life-threatening condition, and patients should be closely monitored while intravenous replacement is initiated.

Autoimmune conditions tend to coexist and should be considered in the differential diagnosis of patients with sustained hypokalemia. Since autoimmune conditions involve multiple systems, thorough investigations should be conducted to arrive at a final diagnosis.

## 8. Conclusion

Clinicians should have a high index of suspicion when a case of chronic hypokalemia presents, even with little to no symptoms suggestive of any other systemic disorder.

## 9. Limitations

It is important to note that the present study was based on a single case report, which may not be generalizable to all individuals with chronic hypokalemia or SLE. Additionally, the underlying mechanisms and causal pathways linking these 2 conditions are not yet fully understood, and further research is required to elucidate the complex interplay between electrolyte disturbances and autoimmune disorders.

This case report represents the experience of a single patient and may not be generalizable. Patient also was on a short follow up which may not be generalizable to the entire population. The diagnosis of LN was not confirmed histologically, as the patient declined a renal biopsy. Nonetheless, clinical and serological findings strongly support the diagnosis of early SLE.

Despite these limitations, the case report discussed in this paper serves as a valuable starting point for exploring the potential connection between chronic hypokalemia and SLE, highlighting the need for greater awareness and investigation in this area.

## 10. Patients perspective

“I have been experiencing recurrent health issues for the past 4 years. Despite seeking care at various hospitals across Bangalore, my symptoms always returned within a few months. Eventually, I was referred to the Shri Atal Bihari Vajpayee Medical College and Research Institution, where I received treatment under the care of Dr Chetan Khoshoo. He expressed genuine concern for my persistent problems, conducted an extensive evaluation, and ordered multiple tests to determine the root cause. Subsequently, I continued my treatment at Dr B.R. Ambedkar Medical College.

Currently, I am on the prescribed medication and feel significantly better. My family and I are deeply thankful to the dedicated doctors and staff at both institutions, whose efforts have greatly improved my quality of life.

## Acknowledgments

I am thankful to the faculty and members at Shri Atal Bihari Vajpayee Medical College and Research Institution for allowing me to work on this case and for guiding me throughout the process. I am also thankful to my parent institution – Dr B.R. Ambedkar Medical College and Hospital for helping me by providing me with directions on how to process this case report.

## Author contributions

**Conceptualization:** Chetan Khoshoo, Kavya Seenahalli Thimmaiah, Bangalore Raja Shivakumar, Parashuram Bhavimani, Avinash Hanbe Rajanna.

**Data curation:** Chetan Khoshoo, Kavya Seenahalli Thimmaiah, Anoop Marilingegowda, Bangalore Raja Shivakumar, Parashuram Bhavimani.

**Formal analysis:** Chetan Khoshoo, Kavya Seenahalli Thimmaiah, Anoop Marilingegowda, Bangalore Raja Shivakumar, Parashuram Bhavimani.

**Investigation:** Chetan Khoshoo, Kavya Seenahalli Thimmaiah, Bangalore Raja Shivakumar, Avinash Hanbe Rajanna.

**Methodology:** Chetan Khoshoo, Kavya Seenahalli Thimmaiah, Anoop Marilingegowda, Bangalore Raja Shivakumar, Parashuram Bhavimani, Avinash Hanbe Rajanna.

**Project administration:** Chetan Khoshoo, Bangalore Raja Shivakumar, Parashuram Bhavimani, Avinash Hanbe Rajanna.

**Resources:** Chetan Khoshoo, Kavya Seenahalli Thimmaiah, Bangalore Raja Shivakumar, Parashuram Bhavimani.

**Software:** Chetan Khoshoo.

**Supervision:** Chetan Khoshoo, Kavya Seenahalli Thimmaiah, Anoop Marilingegowda, Bangalore Raja Shivakumar, Parashuram Bhavimani, Avinash Hanbe Rajanna.

**Validation:** Chetan Khoshoo, Kavya Seenahalli Thimmaiah, Anoop Marilingegowda, Bangalore Raja Shivakumar, Parashuram Bhavimani.

**Visualization:** Chetan Khoshoo, Kavya Seenahalli Thimmaiah, Anoop Marilingegowda, Bangalore Raja Shivakumar, Parashuram Bhavimani, Avinash Hanbe Rajanna.

**Writing – original draft:** Chetan Khoshoo, Avinash Hanbe Rajanna.

**Writing – review & editing:** Chetan Khoshoo, Kavya Seenahalli Thimmaiah, Anoop Marilingegowda, Bangalore Raja Shivakumar, Parashuram Bhavimani, Avinash Hanbe Rajanna.
